# Phase Differences in Expression of Circadian Clock Genes in the Central Nucleus of the Amygdala, Dentate Gyrus, and Suprachiasmatic Nucleus in the Rat

**DOI:** 10.1371/journal.pone.0103309

**Published:** 2014-07-28

**Authors:** Valerie L. Harbour, Yuval Weigl, Barry Robinson, Shimon Amir

**Affiliations:** Center for Studies in Behavioral Neurobiology/Groupe de Recherche en Neurobiologie Comportementale, Department of Psychology, Concordia University, Montreal, QC, Canada; Pennsylvania State University, United States of America

## Abstract

We performed a high temporal resolution analysis of the transcript level of two core clock genes, *Period2 (Per2)* and *Bmal1*, and a clock output gene, *Dbp*, in the suprachiasmatic nucleus (SCN), the master circadian clock, and in two forebrain regions, the lateral part of the central nucleus of the amygdala (CEAl), and dentate gyrus (DG), in rats. These regions, as we have shown previously, exhibit opposite rhythms in expression of the core clock protein, PERIOD2 (PER2). We found that the expression of *Per2*, *Bmal1* and *Dbp* follow a diurnal rhythm in all three regions but the phase and amplitude of the rhythms of each gene vary across regions, revealing important regional differences in temporal dynamics underlying local daily rhythm generation in the mammalian forebrain. These findings underscore the complex temporal organization of subordinate circadian oscillators in the forebrain and raise interesting questions about the functional connection of these oscillators with the master SCN clock.

## Introduction

Circadian rhythms help organisms adapt to their cyclic environment and are important to health in humans. These rhythms are driven by a master clock located in the suprachiasmatic nucleus (SCN) and by subordinate clocks distributed throughout the rest of the brain and body [1]. At the cellular level, the circadian clock is based on transcriptional and posttranscriptional feedback loops driven by protein products of a small set of core clock genes [1], [2]. Using immunohistochemistry, we have previously identified significant daily rhythms in expression of the circadian clock protein, PER2 in multiple forebrain structures including different subregions of the amygdala, hippocampus and cortex in rats [3]. We found, surprisingly, that the PER2 rhythms in the forebrain fall into several different phase clusters all distinct from the phase of the PER2 rhythm of the SCN. Particularly intriguing was the finding that of the different forebrain regions studied, the lateral part of central nucleus of the amygdala (CEAl) and the oval nucleus of the bed nucleus of the stria terminalis (BNSTov), which together form the central extended amygdala, exhibited daily PER2 rhythms that, uniquely, were in antiphase with the rhythms in most other structures, and in close phase with the rhythm of the SCN [3]–[5]. While the functional significance of these divergent PER2 rhythms is yet to be determined, these results lend support to the idea that circadian rhythms in the forebrain are attended by region specific subordinate oscillators driven by differently phased oscillations of clock genes.

The CEAl and dentate gyrus of the hippocampus (DG) exhibit oppositely phased PER2 rhythms, with the rhythm in the CEAl peaking in the evening, in close synchrony with the rhythm of the SCN, whereas the rhythm in the DG peaks in the morning, in antiphase with the rhythm in the CEAl and SCN [3], [5]. In the present study, we sought to determine whether the antiphase PER2 rhythms in the CEAl and DG are attended by similarly opposite rhythms of Per2. In addition we wanted to determine whether the daily rhythm of another essential core clock gene, Bmal1, and of Dbp (albumin D-element binding protein), a clock controlled gene often used as molecular marker of circadian clock output [6], [7], follow similar divergent patterns in the CEAl and DG. We found regional differences in time of peak expression and amplitude of each gene, as well as differences in the phase relationship between the rhythms of Per2 mRNA and PER2 protein, as established in the same rats and reported in a recent paper [3]. These results show that the CEAl and DG exhibit antiphase oscillations at the level of gene transcription and suggest that circadian gene oscillations in the SCN, CEAl and DG involve different molecular dynamics, underscoring the complex temporal organization of subordinate circadian oscillators in the forebrain and their functional connection with the master clock in the SCN.

## Methods

### Animals and tissue preparation

All experimental procedures followed the guidelines of the Canadian Council on Animal Care and were approved by the Animal Care Committee of Concordia University. The current analysis was carried out on brain sections from 74 male Lewis (LEW/Crl) rats used in our previous study of PER2 rhythms in the forebrain [3] and in a study of the rhythms of expression of Per2, Bmal1 and Dbp in the olfactory bulb and in the periphery [8]. As previously described, the rats were individually housed and entrained to a 12 h∶12 h LD cycle, and had free access to food and water. At the appropriate time, they were anaesthetized with sodium pentobarbital (∼100 mg/kg, i.p.) and perfused transcardially with 300 ml of cold saline followed by 300 ml of cold paraformaldehyde (4% in a 0.1 M phosphate buffer, pH 7.3), every 30 min around the 24-h day. The brains were removed, post-fixed overnight in paraformaldehyde at 4°C, and serial coronal sections (50 µm thick) were obtained using a Vibratome (St-Louis, MO). All sections were stored in Watson’s Cryoprotectant at –20°C until use. One set was used for immunohistochemical analysis of PER2 expression as previously reported. A second set was processed in the present study for quantitative real-time polymerase chain reaction (qRT-PCR).

### RNA extraction

Analysis was carried out in seven runs each containing brain samples from 10–11 rats perfused at times distributed randomly across the 24-h day. Free-floating sections were rinsed (6×5 min) in cold TBS to remove the Watson’s Cryoprotectant solution and placed in sterile plastic Petri dishes filled with cold TBS. The three regions of interest (the SCN, CEAl, and DG) were identified using Brain Maps: Structure of the rat brain [9] and bilaterally dissected from the 50 µm thick sections under a dissecting microscope using a scalpel. All instruments were pre-cleaned with RNaseZap solution (#AM9780, Ambion) to neutralize RNase. A new Petri dish, scalpel blade, and gloves were used for each individual brain to avoid contamination. Total RNA was isolated from each of the regions using the RecoverAll™ Total Nucleic Acid Isolation kit (#AM1975, Ambion) following modified manufacturer’s instructions. Briefly, the dissected tissues were dehydrated with two washes in 100% ethanol. The pellet was then air dried at room temperature for 30 min then re-suspended in 150 µl of digestion buffer with 4 µl protease and incubated at 50°C for 15 min and then 80°C for 30 min. RNA was isolated by capture on a glass-fiber filter and purified from residual cellular fragments and proteins by subsequent 100% ethanol and isolation additive washes and centrifugation steps (10,000 *g* for 30 sec). Then, 60 µl of DNase mix was added to the filter and incubated at room temperature for 30 min and washed out with a series of ethanol washes and centrifugation steps (10,000 *g* for 30–60 sec) in order to purify the RNA from DNA residuals. Finally, the purified RNA was eluted in 60 µl of elution solution at room temperature for 1 min and centrifuged at 10,000 *g* for 1 min. Three µl from each sample were taken for RNA quantification and the rest was frozen at −80°C.

The RNA integrity profile (RIN) of each sample and its concentration was assessed using the Experion RNA StdSens Analysis kit (Bio-Rad, Hercules, CA). The Nanodrop2100c spectrophotometer (Thermo Fisher Scientific, Wilmington, DE) was used to measure the absorbance ratios at 260/280 nm and 260/230 nm to assess DNA and protein contamination, respectively. If the absorbance ratio at 260/280 nm was below 1.7, indicating probable DNA contamination, RNA samples were treated with the TURBO DNA-free kit (#AM1907, Ambion) following manufacturer’s instructions. The RNA samples were then re-frozen at −80°C until reverse-transcribed.

### cDNA reverse transcription

Total RNA (≤1 µg) from each brain region of each rat was reverse transcribed to single stranded cDNA using the High-Capacity cDNA Reverse Transcription kit with RNase inhibitor (#4374966, Applied Biosystems, Foster City, CA) according to manufacturer’s instructions. Briefly, the reaction was carried out in a 40 µl volume, consisting of 20 µl of RNA and 20 µl of 2XRT master mix containing 4 µl reverse transcription (RT) buffer, 1.6 µl of 100 mM deoxyribonucleotide triphosphate (dNTP) mixture, 4 µl of 10X random primers, 2 µl of MultiScribe RT enzyme (50 U/µl), and 2 µl of RNase inhibitor. For each brain region, two negative controls were also included: one without the reverse transcriptase enzyme (No Reverse Transcriptase, NRT) to control for any potential genomic DNA contamination, and one without any RNA to assess purity of reagents. Reverse transcription was carried out on the 40-µl samples using the CFX96 Real-Time PCR C-1000™ Thermo Cycler (Bio-Rad) with the following reaction conditions: 10 min at 25°C, 120 min at 37°C, and 5 min at 85°C (enzyme inactivation and denaturation). Once reverse-transcription was complete, samples were stored at −20°C.

### Quantitative Real-Time PCR

For each of the brain structures, mRNA levels for the three target genes (*Per2*, *Bmal1*, and *Dbp)* were determined using qRT-PCR with custom-designed PerfectProbe Gene Detection kits (PrimerDesign, Southampton, UK). Primers/probe sequences and amplicon lengths of the genes of interest are listed in Table 1. The relative quantity of mRNA for each gene of interest was measured relative to four housekeeping genes (HKGs): *B2M* (beta-2-microglobulin), *Hmbs* (hydroxymethyl-bilane synthase), *Top1* (topoisomerase (DNA) 1), and *Ywhaz* (tyrosine 3-monooxygenase/tryptophan 5-monooxygenase activation protein, zeta polypeptide), which were measured using prevalidated PerfectProbe kits (HKG sequences not available, PrimerDesign). The four HKGs used remained stable across the SCN, CEAl, and DG during pre-testing and therefore were deemed to be satisfactory internal controls. Quantitative real-time PCR was performed on the CFX96 Real Time PCR Detection System (Bio-Rad) with the following parameters: Initialization 95°C for 30 sec, followed by 50 cycles of Denaturation 95°C for 10 sec, Annelation 50°C for 20 sec, and Extension (and data collection) 62°C for 30 sec. Amplifications were performed in 20 µl volume reactions containing 5 µl of cDNA (optimally 25 ng), 10 µl of TaqMan Fast Universal Master Mix (#4367846, Applied Biosystems), and 5 µl of PerfectProbe primers/probe mix (PrimerDesign) according to manufacturer’s instructions. Samples with a CT (threshold cycle) value of 35 or less were deemed usable.

**Table 1:** Primer/Probe sequences and Amplicon length of target clock genes.

TargetGene	Sense PrimerSequence, 5′:	Antisense PrimerSequence, 5′:	Probe Sequence, 5′:	Length(bp)
*Per2*	TTC CAC CAG CAA CCC CAA A 3′	CAG GAG TTA TTT CAG AGGCAA GT 3′	CTT CCC CAG CCA GCC TCA CTT TCC Ggg aag 3′	93
*Bmal1*	ACC AGG GTT TGA AGT TAGAGT C 3′	AAG TCA CTG ATT GTG GAGGAA AT 3′	CCA TTC TCT GGT CCG CCA TTG GAA GGg aat gg 3′	88
*Dbp*	ACC CAC TCG CCC AGA CTA TA 3′	AGC AAG CCT CCA GTA TCA GAA 3′	CTT CAA ATC CTA CGA GCA CTG CGG GGG ttg aag 3′	125

For each sample type, a four-fold serial dilution (100–0.39 ng) standard curve was used to determine amplification efficiency of the target genes and the working cDNA concentration, with samples run in duplicate. The qRT-PCR reactions were carried out on 96-well plates. During cDNA synthesis, a negative control (NRT) with all synthesis reagents, but without RNA, was made and run once for every brain region and gene. A no template control (NTC), with water in place of cDNA, was also run for every gene on every plate to rule out contamination. In addition, two positive controls were used in order to validate the efficiency and repetitive nature of the primer/probe assays. Specifically, a commercial brain sample (with validated high RIN quality) was run once for every brain region and gene, and two of the pooled cDNA dilutions taken for the standard curve were run across all plates. All cDNA samples were run in triplicate.

### Data Analysis

The 2^–^ΔΔ^CΤ^ method [10] was applied in order to quantify the relative mRNA levels of *Per2*, *Bmal1*, and *Dbp* for each rat and brain region. First, the target gene expression levels in each rat and brain region were normalized to a combination of the ≥2 most stable HKGs as determined by geNorm software (http://medgen.ugent.be/~jvdesomp/genorm). The relative values were then re-normalized with respect to the highest expression values within each of the seven runs.

**Figure 1:**
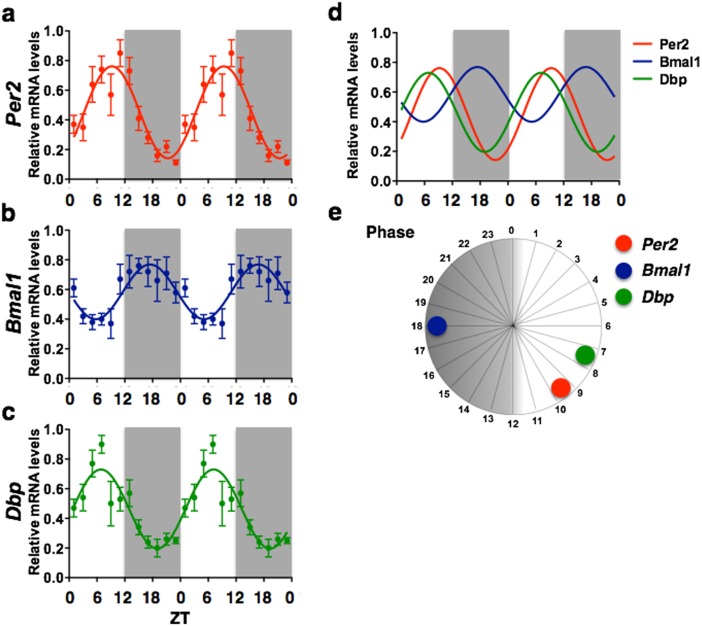
Suprachiasmatic nucleus. Graphs illustrating the temporal organization (a–c), phase relationship (d, e) and relative amplitudes (f) of *Per2*, *Bmal1* and *Dbp* mRNA rhythms in the SCN. The double plotted graphs show relative mRNA levels across 24 *zeitgeber* times fitted with a 24-h sine wave for *Per2* (a), *Bmal1* (b), and *Dbp* (c). Each point represents means ± SEM of mRNA data from 6–7 rats sampled every 30 min and grouped in consecutive 2-h intervals. R^2^ = Goodness of fit value for sine wave. *n* = 73 for all.

**Figure 2:**
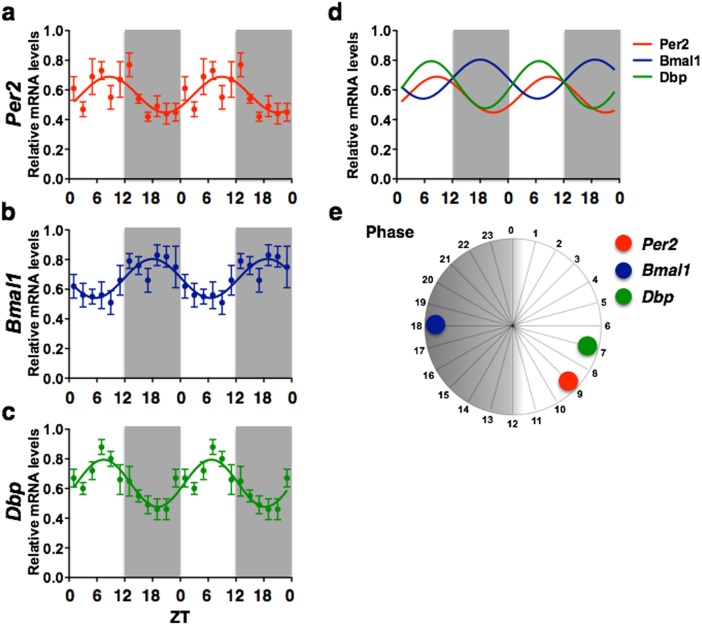
Central amygdala, lateral part. Graphs illustrating the temporal organization (a–c), phase relationship (d, e) and relative amplitudes (f) of *Per2*, *Bmal1* and *Dbp* mRNA rhythms in the CEAl. The double plotted graphs show relative mRNA levels across 12 *zeitgeber* times fitted with a 24-h sine wave for *Per2* (a), *Bmal1* (b), and *Dbp* (c). Each point represents means ± SEM of mRNA data from 6–7 rats sampled every 30 min and grouped in consecutive 2-h intervals. R^2^ = Goodness of fit value for sine wave. *n* = 74 for all.

**Figure 3:**
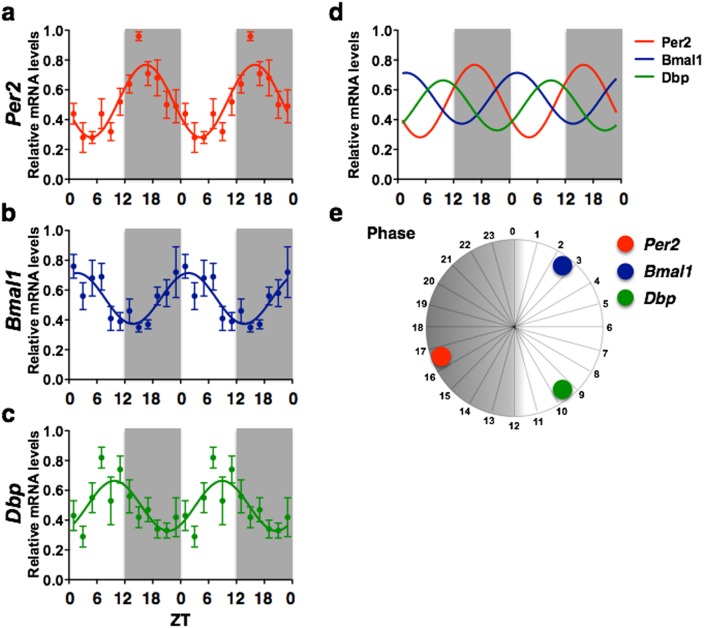
Dentate gyrus. Graphs illustrating the temporal organization (a–c), phase relationship (d, e) and relative amplitudes (f) of *Per2*, *Bmal1* and *Dbp* mRNA rhythms in the DG. The double plotted graphs show relative mRNA levels across 12 *zeitgeber* times fitted with a 24-h sine wave for *Per2* (a), *Bmal1* (b), and *Dbp* (c). Each point represents means ± SEM of mRNA data from 6–7 rats sampled every 30 min and grouped in consecutive 2-h intervals. R^2^ = Goodness of fit value for sine wave. *n* = 74 for *Bmal1* and *Dbp*, *n* = 73 for *Per2*.

**Figure 4:**
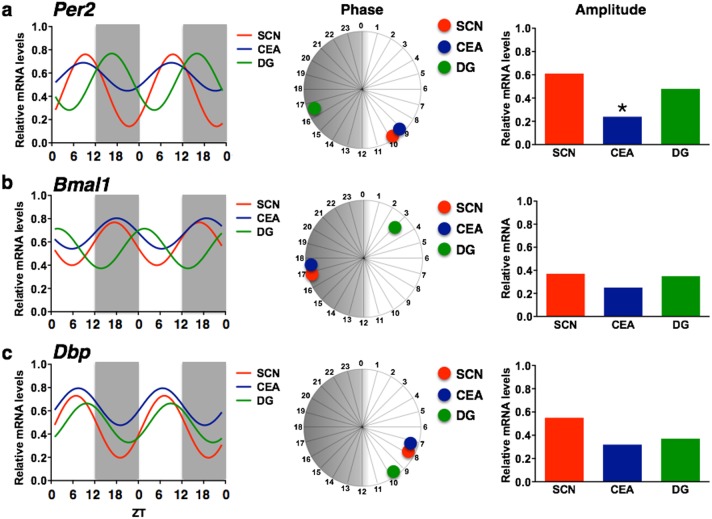
Temporal organization, phase relationship and amplitude of *Per2, Bmal1 and Dbp* expression in the SCN, CEAl and DG. The double plotted line graphs show relative mRNA levels across 12 *zeitgeber* times fitted with a 24-h sine wave between the SCN, CEAl, and DG for *Per2* (a), *Bmal1* (b), and *Dbp* (c). The sine waves were fit to the data grouped in consecutive 2-h intervals as in figures 1–3. The phase relationship between the SCN, CEAl, and DG for each of the genes is depicted in the 24-h circular diagrams and the amplitude between these regions in the bar graphs. Asterisk indicates statistical significance (*p*<.05) as follows: *: CEAl compared to all other regions.

**Figure 5:**
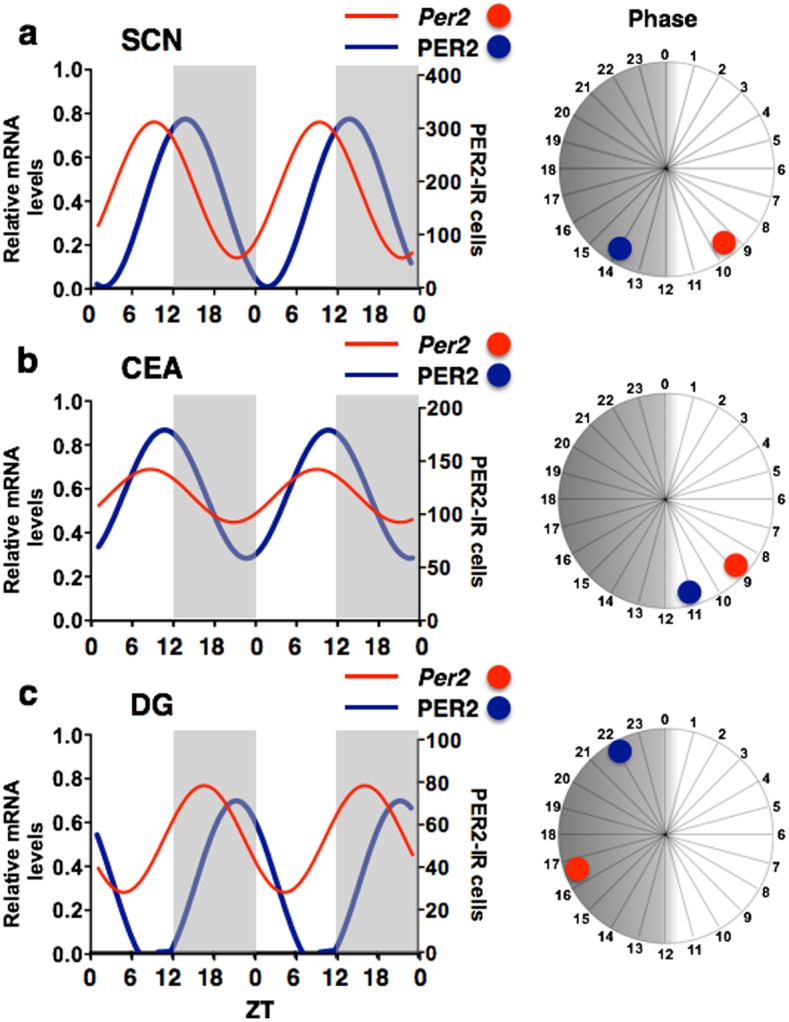
Temporal organization and phase relationship of *Per2* mRNA and PER2 in the SCN, CEAl and DG. The double plotted line graphs show relative *Per2* mRNA (red) and PER2 protein (blue) levels across 12 *zeitgeber* times fitted with a 24-h sine wave in the SCN (a), CEAl (b), and DG (c). The PER2 protein data have been re-plotted from a previously published report using the same rats used in the present study for comparison [3]. The sine waves were fit to the data grouped in consecutive 2-h intervals as in figures 1–3. The 24-h circular diagrams depict the phase relationship between *Per2* mRNA and PER2 protein in each of the three regions analyzed.

Data points for each individual rat were initially single plotted for each brain region and gene using GraphPad Prism (v5.0), with relative mRNA expression on the Y-axis and ZT on the X-axis. The following analysis and statistics were run using the individual data points sampled every 30 min. Sine waves (least-squares regression) with the frequency constrained to exactly 24-h were then fitted to each of these graphs in order to better visualize time of peak and trough for each region and gene using the following equation: Y = M+A*sin (F*X+PS); where M stands for the mesor (i.e. average of the spread of the data from the mid-point of the curve), A stands for amplitude (calculated from the mesor), F stands for frequency (in radians), and PS stands for phase shift, which is the earliest time Y = 0 measured in X axis units. Outliers were determined using the ROUT method with a False Discovery Rate of 1% and removed from all analyses (see [11] for details of the procedure). To further assess the goodness of fit of the curves generated from the sine wave model, the D’Agostino-Pearson omnibus K^2^ normality test [12] was applied to the data. The normality test examines skewness and kurtosis in order to assess how far from Gaussian the data are. A data set fails the normality test when the *p* value is ≤.05, meaning that it deviates significantly from a normal distribution. The statistics for this test are mentioned in the Results section only if they are significant (indicating that the data set has failed the test). Finally, one-way analyses of variance (ANOVA) were used to compare the amplitudes of gene rhythms (established by the sine-fitting model, measured in relative mRNA levels from peak to trough) in the three regions analyzed. Significant effects were further analyzed with Bonferroni multiple comparison post-hoc tests. In order to better visualize the date, the individual data points were grouped into consecutive 2-h intervals and double plotted as shown in figures 1–5.

## Results

We analyzed the expression of the mRNA of *Per2*, *Bmal1* and *Dbp* in the CEAl, DG and SCN in brain sections from 74 rats housed under a 12 h∶12 h LD cycle and perfused at 30-min intervals around the clock. Below we first describe the rhythms of expression of each of the three genes in each region to determine whether the antiphase oscillations in PER2 expression seen previously are mirrored by antiphase oscillations in expression of the clock genes. Next, we compare the rhythms of each gene, in each of the three regions, to highlight phase differences in the expression of any one clock gene across regions. Lastly, we contrast the data on the expression of *Per2* in the SCN, CEAl and DG with data for PER2 reported previously in the same rats to highlight regional differences in the phase relationship between *Per2* and PER2 expression.

The TURBO DNA-free kit was applied to approximately 86% of the SCN samples, 73% of the CEA samples, and 49% of the DG samples to eliminate probable DNA contamination. One sample in the DG *Per2* data set had a CT value of over 35 with high variability between the triplicates and so was excluded from the analysis. No outliers were found in the data using the ROUT method.

### Suprachiasmatic nucleus

The SCN showed a highly rhythmic pattern of *Per2* expression with R^2^ value of 0.496 and amplitude of 0.61 (measured from peak to trough in relative mRNA levels) (Fig. 1a). *Per2* mRNA expression in the SCN peaked at ZT9.5, within the time window previously reported by others [13], [14]. *Bmal1* expression in the SCN also showed a rhythmic pattern, although with a lower R^2^ value (0.297) and lower amplitude (0.37) (Fig. 1b). Peak *Bmal1* expression in the SCN occurs at ZT17.5, preceding the peak in Per2 by about 16 h. The expression of the clock-controlled gene, *Dbp*, was highly rhythmic in the SCN (R^2^ = 0.505) (Fig. 1c) with amplitude of 0.55 and peak expression around ZT7, 14 h after the peak expression of Bmal1. Graphs illustrating the temporal organization and phase relationship of Per2, Bmal1 and Dbp rhythms in the SCN are shown in Fig. 1d and e.

Because the SCN was found to exhibit the most robust mRNA rhythms, the fit (R^2^ value) from the sine wave model for each gene in the SCN was used as the standard for determining the strength of rhythmicity in the CEAl and DG. Given the R^2^ values for the three genes in the SCN, the rhythmicity criteria (1/4th that of the SCN) for the CEAl and DG are as follows: for *Per2* these regions must have an R^2^ value of at least 0.124; for *Bmal1* an R^2^ value of at least 0.074; and for *Dbp* an R^2^ value of at least 0.126.

### Central nucleus of the amygdala

The CEAl exhibited a low amplitude (0.24) rhythm (R^2^ = 0.153) of *Per2* mRNA expression peaking at ZT9 (Fig. 2a). *Bmal1* expression in the CEAl also showed a low relative amplitude (0.25) rhythm (R^2^ = 0.168) with peak expression occurring at ZT18, 15 h before peak Per2 expression (Fig. 2b). *Dbp* expression in the CEAl showed a stronger rhythmic pattern (R^2^ = 0.318) peaking at ZT7, 14 h after peak Bmal1 expression, with relative amplitude of 0.32 (Fig. 2c). Graphs illustrating the temporal organization and phase relationship of *Per2*, *Bmal1* and *Dbp* rhythms in the CEAl are shown in Fig. 2d and e.

### Dentate gyrus

The DG showed a highly rhythmic pattern in *Per2* expression (R^2^ = 0.408) with amplitude of 0.48 (Fig. 3a), however, the 24-h sine wave model failed the normality test (K^2^ = 6.36, *p*<.05), indicating that it did not fit the data perfectly. Peak *Per2* expression in the DG occurred at ZT16.5. *Bmal1* expression also showed a rhythmic pattern (R^2^ = 0.300) peaking at approximately ZT2.5, 14 h before peak expression of Per2, with amplitude of 0.35 (Fig. 3b). Finally, *Dbp* expression in the DG was rhythmic (R^2^ = 0.221) with amplitude of 0.37 (Fig. 3c), peaking at ZT9.5, 7 h after peak expression of Bmal1. Graphs illustrating the temporal organization and phase relationship and relative amplitudes of *Per2*, *Bmal1* and *Dbp* rhythms in the DG are shown in Fig. 1d and e.

### *Per2* in the SCN, CEAl and DG

There are phase differences in *Per2* expression in the three brain regions analyzed. Specifically, *Per2* expression in the SCN and CEAl are virtually in phase, peaking around ZT9, whereas in the DG the Per2 rhythm peaks around ZT16, 7 h later (Fig. 4a). The amplitude of *Per2* mRNA rhythms also vary across regions (*F*_(2,218)_ = 7.4, *p*<.001, Fig. 4a) with the amplitude in the CEAl being significantly lower than in both the SCN and DG (Bonferroni post-hoc tests).

### *Bmal1* in the SCN, CEAl and DG

Phase differences in *Bmal1* expression are also evident between the three regions. Similar to the results obtained for *Per2*, *Bmal1* rhythms in the SCN and CEAl are in phase, peaking around ZT18, whereas in the DG the rhythm peaks about 9 h later, at ZT2.5 (Fig. 4b). The amplitude of the *Bmal1* mRNA rhythms did not differ significantly in these regions (*F*_(2,218)_ = .993, *p* = .372).

### *Dbp* in the SCN, CEAl and DG

The phase differences in peak *Dbp* expression in the SCN, CEAl, and DG are small compared to the differences in phase of peak expression of *Per2* and *Bmal1* in these regions. Specifically, the *Dbp* rhythms seem to cluster, peaking around ZT7 in the CEAl and SCN and about 2 h later, at ZT9, in the DG (Fig. 4c). The amplitude of the *Dbp* mRNA rhythm differs overall between regions (*F*_(2,218)_ = 3.09, *p*<.05), however, Bonferroni post-hoc tests did not find any significant differences (Fig. 4c).

### *Per2* vs. PER2

Finally, we compared the *Per2* data described above with our previously published data for PER2 expression in the SCN, CEAl and DG taken from the same animals [3]. This allowed us to identify regional differences in phase of peak *Per2* mRNA and PER2 protein expression. As shown in Fig. 5, the rhythms of *Per2* expression in the SCN and DG peaked about 5 h before peak PER2 expression, whereas the *Per2* rhythm in the CEAl peaked 2 h before PER2.

## Discussion

Our analysis reveals region-specific differences in the temporal architecture of *Per2* and *Bmal1* mRNA expression in the SCN, CEAl and DG. Specifically, we found that the daily *Per2* and *Bmal1* mRNA rhythms in the CEAl are in phase with the *Per2* and *Bmal1* rhythms of the SCN, and in virtual antiphase with the daily rhythms in the DG. These findings show that the antiphase rhythms in PER2 expression seen in these regions are attended by similar antiphase rhythms in the expression of *Per2* as well as *Bmal1* whose protein, BMAL1, plays a key role in the regulation of *Per2* transcription [2].

Significantly, when we compared the *Per2* rhythms with the rhythms of PER2 protein in each region, we noted regional differences in the time of peak expression of *Per2* mRNA and PER2. In the SCN and DG peak *Per2* mRNA expression preceded that of PER2 by approximately 5 h, consistent with previous findings in the SCN [13]–[15]. Surprisingly, however, the difference between the peak *Per2* mRNA and PER2 expression in the CEAl was only 2 h. This finding points to possible regional differences in the dynamics of *Per2* mRNA translation. Interestingly, in addition to these differences in the temporal organization of the oscillations of clock components, there are other important factors that distinguish between the CEAl and DG oscillations. For example, we have shown previously, in rats, that the PER2 rhythms in the CEAl and DG are differentially affected by manipulations of the external light cycle [16]. Specifically, prolonged exposure to an exotic 26-h LD cycle (1 h∶25 h LD) led to a permanent change in the phase in PER2 expression in the CEAl and to the establishment of a new phase relationship with the PER2 rhythm of the SCN. In contrast, the PER2 rhythm of the DG maintained its normal phase relationship with the SCN. These results suggest that the DG and CEAl oscillators, which have been shown to be under SCN control [5], are differentially sensitive to entraining signals from the SCN or to other internal signals under control of the SCN [16]. Directly related to this idea are the findings that the PER2 rhythms in these two regions are differentially sensitive to steroid hormones. For example, PER2 rhythms in the CEAl and DG are differentially sensitive to endogenous glucocorticoid signalling [17]. Removal of the adrenal glands in rats or genetic deletion of brain glucocorticoid receptors in mice blunts the PER2 rhythm in the CEA [4], [5], [18]. The rhythm is rescued by introducing corticosterone in the drinking water, which establishes an artificial daily rhythm in circulating corticosterone linked to the daily drinking pattern [19]. In contrast, the PER2 rhythm in the DG was not affected by manipulations of circulating corticosterone or brain glucocorticoid receptors, or, additionally, by exogenous administration of corticosterone [20]. Similarly, differential sensitivity of the PER2 rhythms to thyroid hormone signalling was demonstrated following removal of the thyroid and parathyroid glands, which blunted the rhythms in the CEAl but not DG [21]. Moreover, we also found that gonadal hormones selectively modulate the PER2 rhythms in the CEAl [22]. All of these observations indicate the PER2 rhythms in the CEAl (and in many cases the BNSTov) respond uniquely to entraining photic signals from the SCN and to patterns of circulating steroid hormones under the control of the SCN clock. It remains to be shown whether, or how, these differences contribute to the unique pattern of expression of clock genes and proteins in the CEAl (and BNSTov) to set apart from rhythms in most other forebrain regions and in the periphery.

*Dbp* is a clock output gene encoding a PAR leucine zipper transcription factor shown to be involved in various biological processes including circadian locomotor activity rhythms, sleep regulation, hippocampal plasticity, as well as in circadian control of gene expression in peripheral tissues in rodents [7], [23]–[27]. Interestingly, contrary to the large phase difference in *Per2* and *Bmal1* expression rhythms between the SCN, CEAl and DG, there were only small differences in the time of peak expression of *Dbp*. This finding shows that despite differences in the phase of the rhythms of expression of core clock genes (*Per2* and *Bmal1*) and proteins (PER2) in the CEAl and DG, the rhythmic *Dbp* output of these oscillators is coordinated with the rhythmic output of the SCN.

**Figure 6:**
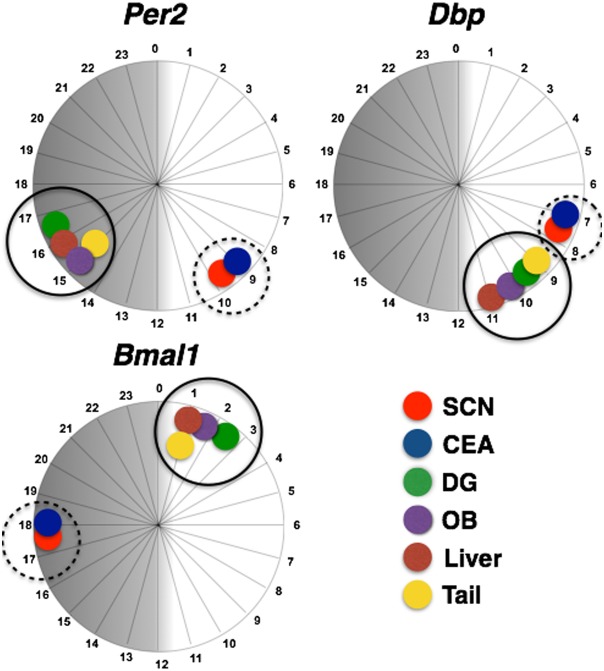
Peak clock gene expression in central and peripheral regions. Twenty-four hour circular diagrams displaying peak *Per2*, *Bmal1* and *Dbp* expression in the SCN, CEAl, DG, olfactory bulb (OB), liver and tail skin. Data for olfactory bulb, liver and skin are based on previously published results [8] using the same rats used in the present study for comparison.

Interestingly, in a previous study using the same rats, we found that the peak expression of *Per2, Bmal1* and *Dbp* are synchronized across the olfactory bulb, liver and tail skin [8]. Furthermore, the rhythms of expression of each of these genes peaks in close temporal proximity with the rhythm in the DG (Fig. 6). Significantly, however, although the temporal architecture of the *Per2* and *Bmal1* rhythms in the three peripheral tissues and DG differ from that of the SCN and CEAl, the rhythms of expression of *Dbp* peak in relatively close temporal proximity across all central and peripheral tissues studied. This finding may point to a mechanism whereby output from differently phased oscillators is coordinated ensuring internal synchrony.

In summary, the present findings reveal both differences and similarities in the timing of expression of core clock components in two forebrain structures, CEAl and DG, shown to exhibit oppositely phased PER2 rhythms in rats. Given that oscillating clock components, including PER2, BMAL1 and DBP operate as transcriptional regulators outside the core clock mechanism, such variations in temporal organization could be translated into differences as well as similarities in local gene expression and ultimately functional output.
